# Griffipavixanthone from *Garcinia oblongifolia* Champ Induces Cell Apoptosis in Human Non-Small-Cell Lung Cancer H520 Cells *in Vitro*

**DOI:** 10.3390/molecules19021422

**Published:** 2014-01-27

**Authors:** Jun-Min Shi, Hui-Juan Huang, Sheng-Xiang Qiu, Shi-Xiu Feng, Xu-E Li

**Affiliations:** 1 School of Life Sciences, South China Normal University, Guangzhou 510631, China; E-Mails: amonolith@126.com (J.-M.S.); hjhuang1980@163.com (H.-J.H.); 2 South China Botanical Garden, Chinese Academy of Sciences, Guangzhou 510650, China; E-Mail: qiusxscbg@163.com; 3 Laboratory of Southern Subtropical Plant Diversity, Shenzhen Fairy Lake Botanical Garden, Chinese Academy of Sciences, Shenzhen 518004, China

**Keywords:** griffipavixanthone, H520 cells, apoptosis, caspase, mitochondrial transmembrane potential, ROS generation

## Abstract

Griffipavixanthone (GPX) is a dimeric xanthone which was isolated in a systematic investigation of *Garcinia oblongifolia* Champ. In this study, we investigate the effect of GPX on cell proliferation and apoptosis on human Non-small-cell lung cancer (NSCLC) cells *in vitro* and determine the mechanisms of its action. GPX inhibited the growth of H520 cells in dose- and time-dependent manners, with IC_50_ values of 3.03 ± 0.21 μM at 48 h. The morphologic characteristics of apoptosis and apoptotic bodies were observed by fluorescence microscope and transmission electron microscope. In addition, Annexin V/PI double staining assay revealed that cells in early stage of apoptosis were significantly increased upon GPX treatment dose-dependently. Rh123 staining assay indicated that GPX reduced the mitochondrial membrane potential. DCFH-DA staining revealed that intracellular ROS increased with GPX treatment. Moreover, GPX cleaved and activated caspase-3. In summary, this study showed that GPX inhibited H520 cell proliferation in dose- and time-dependent manner. Further mechanistic study indicated that GPX induced cell apoptosis through mitochondrial apoptotic pathway accompanying with ROS production. Our results demonstrate the potential application of GPX as an anti-non-small cell lung cancer agent.

## 1. Introduction

Lung cancer is the most common cancer and the major cause of mortality throughout the World. Over one million people worldwide have been diagnosed with this devastating disease, which causes over 400,000 deaths annually. Among them, non-small-cell lung cancers (NSCLC) make up about 85% of all kind of lung cancers [1,2,3]. Conventional therapeutic strategies, including surgery, radiation and chemotherapy, cause clinically significant adverse effects that limited effectiveness [3,4]. Thus, novel chemotherapeutic drugs for treating NSCLC are urgently needed. Epidermal growth factor receptor (EGFR) tyrosine kinase inhibitors such as gefitinib and erlotinib have been developed to treat NSCLC. However, they were only effective in one-third of all non-small cell lung cancer patients. Moreover, drug resistance is commonly seen in these patients [5,6,7,8]. Recently, compounds isolated from medicinal plants, such as vinblastine (*Catharanthus roseus*) and paclitaxel (*Taxus brevifolia*), have shown promising cancer therapy effects [9]. Therefore, systematic investigations on the active principles of the medicinal plants as well as the molecular mechanism would lead to the discovery of novel anti-cancer drugs.

*Garcinia oblongifolia* Champ has long been used as a folk medicine to treat burns and inflammation. In our preliminary screening, the crude extract of *G. oblongifolia* showed anti-tumor potential. In the present study, we conducted bioassay-guided fractionation and purification of *G. oblongifolia* and isolated the major active substance, griffipavixanthone (GPX), which exhibited anti-proliferative effect on H520 cells. Mechanistically, we found that GPX inhibit non-small cell lung cancer cell growth through inducing mitochondrial apoptotic pathway accompanying with ROS production.

## 2. Results

### 2.1. Identification and Anti-Proliferative Effect of GPX on NSCLC Cells

**Figure 1:**
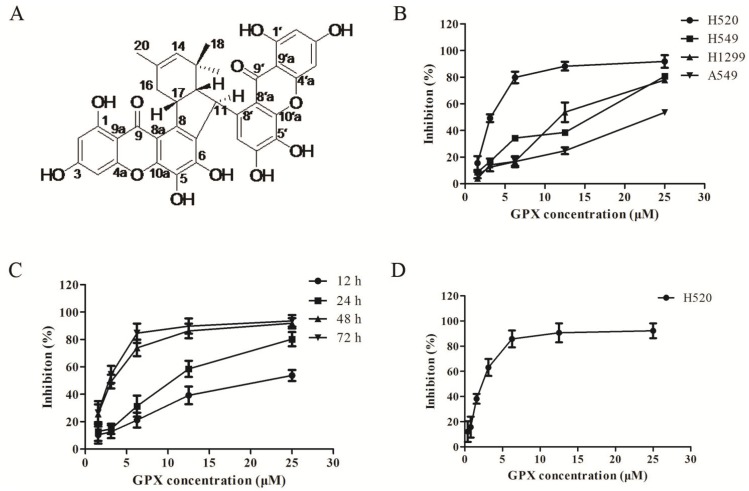
GPX inhibits the growth of NSCLC cells. (**A**) Chemical structure of GPX. (**B**) H520, H549, H1299 and A549 cells were treated with different concentrations of GPX for 48 h. Cell viability was determined by MTT assay. (**C**) H520 cells were treated with different concentrations of GPX for 12, 24, 48 and 72 h. Cell viability was determined by MTT assay. (**D**) H520 cells were treated with different concentrations of GPX for 48 h and Cell viability was determined by NRU assay.

The chemical structure of GPX was identified as shown in Figure 1A according to the previously published data [10,11]. It is a bixanthone in which two xanthones are linked *via* a double cyclization involving two prenyl groups [10,11,12].

To evaluate the anti-proliferative effect, several human non-small cell lung cancer cell lines were treated with various concentrations of GPX for 48 h. As indicated by the cell survival curve, GPX inhibited the survival of H520, H549, H1299 and A549 cells with IC_50_ values of 3.03 ± 0.21, 11.1 ± 0.33, 12.12 ± 0.25 and 29.33 ± 0.51 μM (Figure 1B). Moreover, H520 cells were treated with different concentrations of GPX for 12 h, 24 h, 48 h and 72 h. Cell viability data indicated that GPX inhibited cell growth time-dependently (Figure 1C). Neutral red uptake (NRU) assay further confirmed that GPX inhibited the proliferation of H520 cells in a dose-dependent manner, with IC_50_ values of 1.88 ± 0.36 μM (Figure 1D).

### 2.2. GPX Induced Apoptosis on H520 Cells

**Figure 2:**
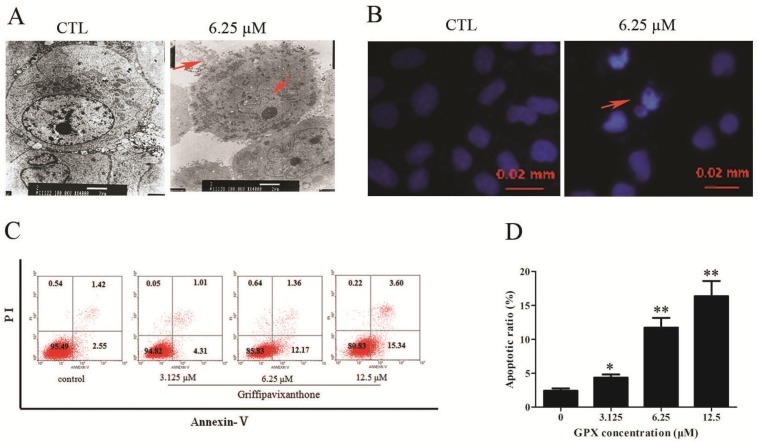
GPX induces apoptosis in H520 cells. (**A**) Induction of apoptosis in H520 cells by GPX as compared to an untreated control morphologically. After 48 h treatment with GPX, morphological changes like shrinking, bright, roundout, vacuolation and rupture of membrane were observed in treated cells by TEM. (**B**) Generation of apoptotic body of H520 cells by GPX treatment for 48 h. (**C**) The H520 cells were treated with indicated concentrations of GPX for 48 h and the Annexin V/PI dual staining was used to detect apoptosis using flow cytometry. (**D**) Apoptotic ratio of H520 cells after GPX treatment at 48 h was detected by Annexin V/PI dual staining assay, * *p* < 0.05 and ** *p* < 0.01.

To access whether GPX induces apoptosis in H520 cells, the morphological changes and phosphatidylserine externalization were assessed. Ultra-structural observation on GPX-treated cells by TEM and Hoechst staining observation showed volume shrinkage, nuclear condensation and apoptosome formation, which both signified GPX-induced apoptosis in H520 cells (Figure 2A,B). Apoptotic cells were further quantified by Annexin V/PI double staining assay. GPX treatment significantly increased the percentage of apoptotic cells (2.55% to 15.34%) in H520 cells (Figure 2C,D). These results suggested that the growth inhibition of GPX was at least in part due to apoptosis of H520 cells.

### 2.3. GPX Induced Apoptosis *via* Mitochondrial Apoptotic Pathway

**Figure 3:**
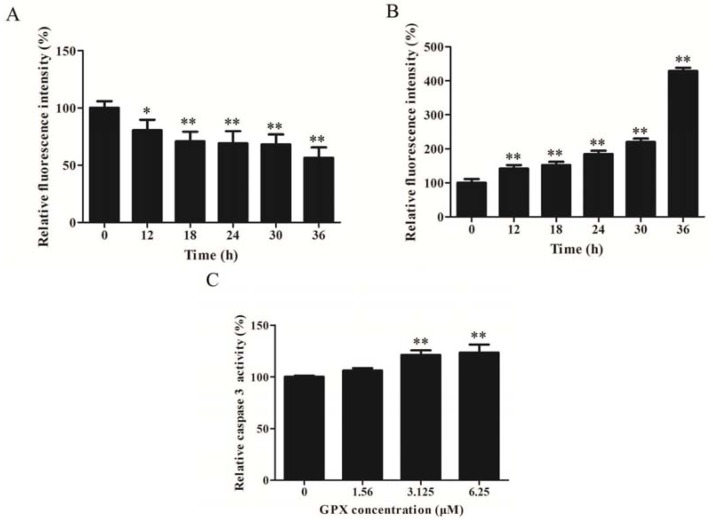
GPX induced apoptosis *via* mitochondrial apoptotic pathway. (**A**) Induction of mitochondrial membrane potential collapse of H520 cells by GPX treatment for indicated time intervals, * *p* < 0.05 and ** *p* < 0.01. (**B**) Induction of intracellular ROS in H520 cells by GPX treatment at different time intervals, * *p* < 0.05 and ** *p* < 0.01. (**C**) Caspase 3 was cleaved and activated by GPX treatment at different concentrations, * *p*< 0.05 and ** *p* < 0.01.

ΔΨm is the hallmark of the status of mitochondrial membrane. As shown in Figure 3A, GPX treatment resulted in a time-dependent loss of ΔΨm, as evidenced by the shift of fluorescence. Moreover, loss of ΔΨm is always closely linked with oxidative stress. Therefore, we examined whether there is more ROS production upon GPX administration. To this end, we performed DCFH-DA-based fluorescence detection by flow cytometry. The results showed that GPX treatment on H520 cells increased ROS levels in a time-dependent manner (Figure 3B). Furthermore, GPX treatment led to cleavage of caspase-3 on H520 cells (Figure 3C).

## 3. Discussion

Uncontrolled cellular growth, as a result of defects in the apoptotic machinery and cell cycle regulation, is responsible for most of cancer progression [9,13]. Therefore, agents what induce cell apoptosis may be effective in cancer therapy. Natural products possessing unique structural properties targeting cell proliferation represents the important sources for the development of novel anti-tumor drugs.

During our *in vitro* on-going screening program of anti-cancer components in Chinese herbs, the 95% ethanol extract of *G. oblongifolia* showed potent inhibitive effect on NSCLC cell growth. Thus, identifying the active chemical entities and elucidating the molecular mechanisms in suppressing tumor cell growth could enable better application of the traditional Chinese medicine in cancer therapy. Xanthones, the major components of *Garcinia* plants, have been reported with antiproliferative effect on various human tumor cell lines [14,15,16,17,18]. The bixanthones, formed by xanthone dimerization, are characterized by unique chemical structure. Studies showed that bixanthones exhibited potent bioactivities and promising pharmacological profiles such as cancer chemopreventive, anthelmintic, antimalarial, antitubercular and so on [19,20,21]. In the present study, we isolated GPX from *G. oblongifolia* by a bioassay and characterized the structure by ESI-MS, ^1^H-NMR, and ^13^C-NMR spectroscopic analyses. GPX possesses cytotoxic activity on murine cancer cells, such as P388 leukemia cells, LL/2 Lewis lung carcinoma cells and Wehil64 sarcoma cells [10], but its effect on human cells and the mechanism is not available. It is very interesting to indicate its medicinal function. The cytotoxic effect of GPX was firstly evaluated on NSCLC cells. GPX treatment inhibited the growth of H520 and other NSCLC cells in a dose-dependent manner detected by MTT assay. Moreover, the NRU assay, which is based on the ability of viable cells to incorporate and bind the supravital dye neutral red in the lysosomes, was used to quantitative estimation of the number of viable cells after GPX treatment, with IC_50_ values of 1.88 ± 0.36 μM. Furthermore, GPX indicated significant anti-proliferative activity on other human cancer cell lines, including human breast cancer cells (MCF-7 and MDA-231), human prostate cells (DU145, PC3 and LNcaP) and human colon cancer cells (HCT-116, HT-29 and SW-480) in a dose dependent manner, with IC_50_ values of 9.36 ± 0.73, 3.88 ± 0.56, 7.93 ± 0.38, 20.91 ± 0.79, 4.31 ± 0.29, 10.52 ± 0.14, 6.86 ± 0.68 and 5.61 ± 0.85 µM, respectively, at 48 h (data not shown). Moreover, GPX had low cytotoxic effects on non-tumorigenic LO2 cells with IC_50_ of 29.08 ± 0.61 µM, indicating that it may be selectively cytotoxic for human cancer cells. Therefore, GPX prospectively becomes a better therapeutic candidate to NSCLC with strong anti-cancer activity and low cytotoxicity to normal cells.

In the present study, we attempt to elucidate the possible mechanisms of GPX-induced cell death on H520 cells. It is exciting to detect whether GPX induced cell death *via* apoptosis on H520 cells. The proportion of cells in the early stage of apoptosis was quantified using Annexin V/PI double staining. We found that the ratios of early apoptotic cells were increased in a dose-dependent manner in H520 cells treated with GPX (Figure 2A). Our results also showed an increase of caspase-3 activity, which is critical in proteolytic cascade within the apoptosis signal pathway [22], in GPX-treated H520 cells (Figure 3C). Mitochondria dysfunction always plays an important role in promoting the activation of caspases. It is believed that impaired permeability across the mitochondria membrane favors membrane hyperpolarization and increasing reactive oxygen species (ROS) production. When H520 cells were treated with GPX, ΔΨm was time-dependent lost and ROS level was time-dependent increased. Induction of ROS may activate MAPK and mTOR pathways which lead to mitochondria-caspase apoptotic pathway. It is very interesting to further study the role of MAPK and mTOR pathways in the GPX-induced apoptosis. All these results suggested that GPX induced cell apoptosis *via* mitochondria-caspase apoptotic pathway.

## 4. Experimental

### 4.1. Reagents and Chemicals

RPMI-1640, DMEM were purchased from Gibco (Carlsbad, CA, USA). Fetal calf serum was purchased from Zhejiang Tianhang Biological Technology (Hangzhou, Zhengjiang, China). Propidium iodide (PI), 3-[4,5-dimethylthiazol-2-yl]-2,5-diphenyltetrazolium bromide (MTT), Rhodamine 123 (Rh123) and DCFH-DA were obtained from Sigma-Aldrich (St Louis, MO, USA). Annexin V-FITC/PI was purchased from BD Bioscience (San Jose, CA, USA). Trypsin, Giemsa, Neutral red dye and trypan blue were purchased from Amresco (Solon, OH, USA). Hochest 33258 was purchased from Merk (Whitehouse Station, NJ, USA). Caspase-3 Activity Assay Kit and Bradford Protein Assay Kit were purchased from Beyotime (Nantong, Jiangsu, China)

### 4.2. Plant Materials

The bark of *G. oblongifolia* was collected in Zhaoqing City (China), and authenticated by Fu-Wu Xing of South China Botanical Garden, Chinese Academy of Sciences. Voucher specimen was deposited in the South China Botanical Garden, Guangzhou, China.

### 4.3. Extraction and Isolation

The air-dried bark (15 kg) was powdered and extracted with 95% EtOH (50 L) for three times at room temperature. The crude extract (1.2 kg) was obtained after solvent removal under reduced pressure. It was dissolved in water and partitioned with petroleum ether and ethyl acetate (EtOAc). The petroleum ether fraction was purified by silica gel column chromatography to obtain a sub-fraction that was chromatographied on silica gel and Sephadex-LH 20 to give GPX. The purity of GPX was determined by high-performance liquid chromatography (HPLC) to be 98%. GPX was dissolved in DMSO to make a 50 mM stock solution and stored at −20 °C. Each working solution was freshly prepared in the cell culture medium with a final DMSO concentration of less than 0.1%.

### 4.4. Cell Culture

H520, H549, H1299 and A549 human non-small cell lung cancer cells were obtained from American Type Culture Collection (Manassas, VA, USA). Cells were grown in DMEM supplemented with 10% fetal bovine serum and 1% (*v/v*) penicillin-streptomycin in a humidified environment with 5% CO_2_ at 37 °C.

### 4.5. MTT Assay

H520, H549, H1299 and A549 cells were seeded in 96-well plates, and then treated with GPX for 48 h. MTT (5 mg/mL) was added into each well and incubated for 4 h. The culture medium was discarded and 100 μL DMSO was added to solubilize the formazan product. The absorbance at 570 nm was recorded and the concentration required to inhibit cell growth by 50% (IC_50_) was calculated from survival curves.

### 4.6. NRU Assay

H520 cells were seeded in 96-well plates and exposed to GPX for 48 h. Then cells were incubated with 0.5 mg/mL Neutral red for 30 min. Cells were rinsed with PBS twice and lysed with 100 μL acetic acid: ethanol: water (1: 50: 49) for 20 min. The absorbance at 570 nm was recorded. IC_50_ was calculated from survival curves.

### 4.7. Hoechst 33,258 Staining

H520 cells were fixed after treated with GPX for 48 h, and then stained with 10 μg/mL Hoechst 33258 for 10 min. The cells were gently washed with PBS and photographed by a ZEISS AXIOPLAN2 fluorescence microscope (Jena, Germany).

### 4.8. Transmission Electron Microscope Analysis

H520 cells were obtained after treatment with GPX for 48 h, fixed with glutaraldehyde and osmic acid, dehydrated with ethanol, and then stained with uranyl acetate and lead citrate. The ultra structure was analyzed with a JEM-1010 transmission electron microscope (JEOL, Tokyo, Japan).

### 4.9. Cell Apoptosis Analysis

H520 cells were treated with GPX for 48 h before harvested, then stained with 2 μL Annexin V-FITC and 5 μL PI for 10 min. After treatment, viable cells are Annexin V-FITC and PI negative, early apoptosis cells are Annexin V-FITC positive and PI negative, late apoptosis or already dead cells are both Annexin V-FITC and PI positive. Cells were analyzed by flow cytometry.

### 4.10. Mitochondrial Membrane Potential (ΔΨm) Assay and Intracellular ROS Assay

H520 cells were treated with 6.25 μM GPX for 12 h, 18 h, 30 h and 36 h, respectively. Afterwards, cells were collected and incubated with 1 μM Rhodamine 123 at room temperature for 30 min or 1 μM DCFH-DA at 37 °C for 30 min. The fluorescence was detected by flow cytometry.

### 4.11. Caspase-3 Activity Assay

H520 cells were treated with GPX for 48 h and lysed in 100 μL lysis buffer. Bradford Protein Assay Kit was used to measure protein concentration. Then caspase-3 activity was measured with a Caspase-3 Activity Assay Kit according to the manufacturer’s instructions.

### 4.12. Statistical Analysis of Biological Assays

All results are presented as means ± standard deviation from triplicate experiments performed in a parallel manner. Statistical differences were determined using the Student’s t-test. All comparisons are made relative to untreated controls. A statistically significant difference was considered at * *p* < 0.05 and ** *p* < 0.01.

## 5. Conclusions

In summary, this is the first study of the isolation GPX with potential anti NSCLC activity from *G. oblongifolia*, and a mechanistic study on the mechanisms of its cytotoxic activity. Our data indicate that GPX has anti-proliferative effect on human NSCLC cells *via* cell apoptosis. Furthermore, our findings suggest that GPX induced apoptosis *via* caspase activation and mitochondria dysfunction. Taken together, these results will contribute to the development of GPX into a new chemotherapeutic agent for the treatment of NSCLC.
